# High Seropositivity of Markers of Viral Infections among Women with Unfavorable Pregnancy Outcomes in Mwanza, Tanzania: The Urgent Need for Control Interventions

**DOI:** 10.24248/eahrj.v7i1.705

**Published:** 2023-07-12

**Authors:** Ulimbakisye Mcdonald, Helmut Nyawale, Alphaxard Kajura, Fridolin Mujuni, Elieza Chibwe, Vitus Silago, Betrand Msemwa, Caroline A Minja, Zawadi Daffa, Mahmud Karim, Evidence C. Byasharila, Alda Ester Chongo, Stephen E. Mshana, Mariam M. Mirambo

**Affiliations:** a Department of Obstetrics and Gynecology, Weill Bugando School of Medicine, Catholic University of Health and Allied sciences, Mwanza, Tanzania; b Department of Microbiology and Immunology, Weill Bugando School of Medicine, Catholic University of Health and Allied Sciences, Mwanza, Tanzania; c Institute of Allied Health Sciences, Catholic University of Health and Allied sciences, Mwanza, Tanzania; d Department of Biochemistry and Molecular Biology, Weill Bugando School of Medicine, Catholic University of Health and Allied sciences, Mwanza, Tanzania; d Department of Biological Sciences, Eduardo Mondlane University, Maputo, Mozambique

## Abstract

**Background:**

Viral infections such as Human cytomegalovirus (HCMV), Rubella virus (RV) and Herpes simplex virus-2(HSV-2) are implicated in causing adverse pregnancy outcomes with limited data from Africa. Here we report the magnitude of these viruses among women with unfavorable pregnancy outcomes (WUP) in Mwanza, Tanzania.

**Methods:**

A cross sectional study involving 198 WUP was conducted between March and June 2019 in Mwanza. Enzyme linked immunosorbent assay was used to detect HCMV and RV IgM and IgG antibodies while immunochromatographic test was used to detect HSV-2 IgM and IgG antibodies. Data were analyzed by using STATA version13.0.

**Results:**

The median age of enrolled women was 28(IQR, 24-34) years. Of these 194(98%) were HCMV IgG seropositive while only 2(2.1%) were IgM seropositive. Out of 180 women tested for RV, 175(96.7%) were IgG seropositive while only 1(1.2%) was RV IgM seropositive. Regarding HSV2; out of the 146 women tested, 21(14.4%) were seropositive for HSV2 IgG, and only 3(2.1%) were HSV-2 IgM seropositive. Having primary education (*p=.046*) and being married (*p=.035*) were significantly associated with HSV-2 IgG seropositivity.

**Conclusion:**

A substantial proportion of WUP have markers of viral infections for potential pathogens that might be associated with unfavorable pregnancy outcomes necessitating further studies to establish causal effect relationship.

## BACKGROUND

Previous unfavorable pregnancy outcome implies previous adverse fetal outcome in terms of two or more consecutive spontaneous abortions, history of fetal death, intrauterine growth retardation, still birth, early neonatal death and/or congenital anomalies.^1^ The human cytomegalovirus (HCMV), Rubella virus (RV) and herpes simplex type 2 virus (HSV-2) are the common pathogens causing congenital infections worldwide. HCMV infection during pregnancy is far more complex than other infections due to the ability of the virus to be frequently reactivated during the child bearing age and be transmitted to the fetus in spite of maternal immunity.^2^

HCMV rarely causes disease in healthy individuals. However; it can result into spontaneous abortion, intrauterine fetal death (IUFD), still birth, early neonatal death or congenital anomalies.^1,3^ The seropositivity rate for HCMV has been found to range from 14.2% in Iran, to 91.05% in India in women with unfavorable pregnancy outcomes.^4, 5^ In Africa among pregnant women, the HCMV prevalence of 96% and >73.95% has been reported in Egypt and Tanzania respectively.^6 7 8^ On the other side, RV infection in early pregnancy has been associated with adverse pregnancy outcomes such as abortions, stillbirths and congenital Rubella syndrome (CRS). In high-income countries where strategic immunization programs such as Rubella immunization, has been implemented, the number of CRS cases have been widely reduced.^9^ However, it remains a public health problem in most of resource constrained countries particularly in the sub-Saharan Africa.^10^ Regarding Herpes simplex virus type 2(HSV2), vertical transmission with HSV2 often results into neonatal herpes. Neonatal herpes is a serious condition in newborns with an estimated incidence of 10 per 100,000 live births globally.^11–14^

Different viral pathophysiological mechanisms have been documented to cause negative effects to the newborns, one being upregulation of cytokines and interferons in infected chorion epithelium and endothelia cells which could contribute to congenital defects. The other one is through inhibition of intracellular assembly of the chorionic epithelium cells which lead to inhibition of mitosis and restrict development of precursor cells for fetal development.^15^

Screening of these viruses is not included in the current antenatal package in most of low and middle-income countries. A previous study in Mwanza showed high HCMV IgG seropositivity which was linked with previous history of abortion. The study also observed that all women with history of stillbirth and having baby with congenital malformation were HCMV IgG seropositive.^8^ On the other side adverse pregnancy outcomes associated with RV has been reported in this setting.^16^ This implies that these viruses might significantly contributes to unfavorable pregnancy outcomes in this setting. In a view of that this study was conducted to establish the magnitude of these infections among women with previous unfavorable fetal outcomes. This information may create public awareness and influence policy makers to consider formulating a policy of screening for these viruses prior to or during pregnancy and treating those with severe complications to prevent adverse pregnancy outcomes.

## METHODS

### Study Design and Study Area

Between March 2019 and June 2019 an analytical cross-sectional hospital-based study was conducted from three selected health facilities in Mwanza. The study was carried out at clinics and postnatal/gynecology wards of the department of Obstetrics and Gynecology at Bugando Medical Centre (BMC-49), Sekou Toure Regional Referral Hospital (SRRH-60) and Sengerema DDH (SDDH-89). Conveniently we selected the BMC which is a tertiary zonal hospital serving an estimated population of 18 Million people^17^ and SSRH to represent urban population. In addition, SDDH was selected to represent rural population.

BMC is a consultant/referral and teaching hospital for the Lake and Western zones of the United Republic of Tanzania. It is situated along the shores of Lake Victoria in Mwanza City; it has 947 bed capacity and has approximately 450 numbers of deliveries per month and more than 311 women with previous unfavorable fetal outcomes of unknown cause for the past one year (January 2017 to March 2018). SRRH is a regional referral hospital located in North western part of Tanzania along the shores of the Lake Victoria in Mwanza city. It has 375 bed capacity, with approximately 700 numbers of deliveries per month and more than 140 cases of previous unfavorable fetal outcomes reported in three months according to register book (Mfumo wa Taarifa za Uendeshaji Huduma za Afya-MTUHA). SDDH is located in a rural area with an estimated population size of 663,034, bed capacity of 320 beds, approximately 750 deliveries, and more than 130 cases of previous unfavorable fetal outcomes in three months (MTUHA register book).^17^

### Study Population, Eligibility Criteria and Sampling Techniques

The study included women with previous unfavorable pregnancy outcomes which occurred not more than two years attending at BMC, SRRH and SDDH for either antenatal or maternity/postnatal services during the study period. The study included all non-pregnant women with previous unfavorable pregnancy outcomes that occurred within two years and women with established cause of unfavorable pregnancy outcomes such as DM, thyroid disease, syphilis, hypertension, severe anemia, SCD and antiphospholipid syndrome were excluded.

Sample size (n) was calculated using Kish Lisle formula (1965) for cross sectional studies using the prevalence of 14.2% from previous study^4^ whereby a total of 187 was obtained. However, a total of 198 women were enrolled. Convenient sampling of patients who met the inclusion criteria was performed until the sample size was reached. Socio-demographic data and blood samples were collected from 198 consenting participants.

### Sample Collection and Laboratory Procedures

About 4 - 5 ml of blood was drawn aseptically from median cubital vein from each consented participant and kept in plain sterile vacutainer tubes (Becton Dickinson LTD, Nairobi, Kenya). The tubes were kept at upright position at temperature 21-25 C° and then transported to CUHAS microbiology laboratory. Sera were extracted, stored in cryovials and kept at -80^0^C until processing. Before analysis sera were removed from a deep freezer and left at room temperature for 20 to30 minutes. HCMV and RV antibodies were analysed by indirect enzyme linked immunosorbent assay (ELISA) as per manufacturer's instructions (Qingdao Hightop Biotech Co.Ltd, China) with sensitivity and specificity of > 99%. HSV2 antibodies were detected by using rapid immunochromatographic test as per manufacturer's instructions (Exact Diagnostic Devices-USA). This test has a sensitivity of 95% and specificity of 94.7%. All tests were performed following the guidelines written by the manufacturer; the quality control (QC) directives were followed accordingly.

### Study Variables and Data Analysis

Dependent (outcome) variables was seropositivity of HCMV, RV and HSV2 while independent variables included socio-demographic characteristics (age, marital status, educational level, occupation), maternal characteristics (gravidity, parity, gestation age) miscarriage, stillbirth, prematurity, organ transplant and other health system related characteristics (antenatal care attendance, booking place) number of family member (family size), type of house, type of toilet, source of drinking water, history of illness in the previous pregnancy.

Data was double entered, verified and cleaned using Excel 2007. Statistical data analysis was done using STATA version 13.0 (StataCorp LP, College Station, TX, USA. Categorical variables such as residence, socioeconomic status, blood transfusion and past obstetrics history such as history of miscarriage, history of stillbirth, and preterm delivery were described as proportions whereas continuous variables like age, household members were summarized as medians (interquartile range). The variables with a p-value of less than 0.05 were considered statistically significant. Socioeconomic status (SES) was determined by house type, toilet type and source of water whereby high socioeconomic status was defined by having modern house, modern toilet and using tap water.

### Ethical Considerations

Ethical clearance was sought from the joint Catholic University of Health and Allied Sciences/Bugando Medical Centre (CUHAS/BMC) Ethics and Review committee (CREC) with ethical clearance number CREC/346/2019. Permission was requested from relevant governmental and hospital's authorities. A written informed consent was requested from the participants after explaining the aims of the study. In this study we enrolled women above 16 years, as per Tanzania ethical guidelines, an individual above this age is allowed to sign a written informed consent.

## RESULTS

### Socio-Demographic Characteristics of Enrolled Women

A total of 198 women with unfavorable previous pregnancy outcome were recruited in this study. The median age of study participants was 28(IQR 24-34) years. Among these women, 89(44.95%) and 109 (55.05%) were from rural and urban areas, respectively. The median number of household members was 5(IQR: 4-7). The majority of them 167 (84.34%) were married while two thirds 134 (67.68%) had primary education level. More than a half 118(59.6%) were found to have low socioeconomic status (SES) (Table 1).

**TABLE 1:** Distribution of Socio-Demographic Characteristics Of 198 Women With Unfavourable Previous Pregnancy Outcomes Enrolled In Mwanza Region, Tanzania

Characteristics	Number (n)	Percentage (%)/Median
**Age (years)	198	28[IQR24–34]
Education		
Primary	134	67.6
Secondary	36	18.2
College	14	7.10
No education	14	7.1
Occupation		
Peasant	43	21.7
Employed	15	7.6
Business	80	40.4
House wife	60	30.3
Residence		
Rural	89	44.9
Urban	109	55.1
*SES		
High	80	40.4
Low	118	59.6
Marital status		
Married	167	84.4
Single	31	15.6
**Family members	5	4–7

* SES= socioeconomic status,

** continuous variables summarised as median

### Clinical Characteristics and Previous History of Unfavourable Pregnancy Outcomes in Mwanza

Among 198 women with previous unfavorable pregnancy outcomes, 193(97.5 %) had history of abortion and out of these 186 (94%) experienced 1-3 abortions. A considerable proportion 42(21.2%) of these women had history of blood transfusion. About one third 64(32.3%) had history of preterm birth while more than half 114(58.0%) had history of stillbirth. It was also noted that 2(1.0%) women had history of delivering children with congenital anomalies (Table 2).

**TABLE 2:** Clinical Characteristics and Previous History Among Women with Previous Unfavorable Pregnancy Outcomes in Mwanza

Characteristics (variables)	Frequency (n)	Percent (%)
Abortions		
No	5	2.5
Yes	193	97.5
Number of abortions		
0	5	2.50
1–3	186	93.9
> 3	7	3.6
Preterm birth (baby)		
No	134	67.7
Yes	64	32.3
No of preterm birth		
0	134	67.7
1	57	28.7
>2	7	3.5
Stillbirth		
No	84	42.4
Yes	114	57.6
No of stillbirth (IUFD)		
0	84	42.4
1	86	43.4
2	26	13.1
3	2	1.1
Rash		
No	194	98.0
Yes	4	2.0
H/baby with disability		
No	196	98.9
Yes	2	1.0
H/Blood transfusion		
No	156	78.8
Yes	42	21.2
HIV status		
Negative	188	94.9
Unknown	10	5.0

### Seropositivity of HCMV, RV and HSV2, and Associated Factors Among Women with Previous Unfavourable Pregnancy Outcomes in Mwanza, Tanzania

Among 198 women with previous unfavourable pregnancy outcomes tested, 194 (98%, 95% CI: 96.0-99.9) were found to be HCMV IgG seropositive while only 2(2.1%. 95% CI: 0.8-5.1) were IgM seropositive. Out of 180 women who tested for RV antibodies only 1(1.2%, 95% Cl 1.1-3.5) tested positive for RV IgM antibodies while 175(96.7%, 95% cl 94.1-99.3) tested positive for RV IgG antibodies.

Regarding HSV2, out of 146 women tested for HSV2 antibodies, 21(14.4%, 95% CI: 8.6-20.0) tested positive for HSV-2 IgG antibodies and 3(2.05%, 95% CI: 0.2-4.3) tested positive for HSV-2 IgM antibodies.

Primary level of education (X^2^ =6.15, p=0.046) and being married (fishers exact=0.079, p=0.035) were significantly associated with HSV-2 IgG seropositivity while factors associated with HCMV and RV seropositivity could not be investigated because almost all had the outcome of interest. The two women who were HCMV IgM seropositive; had history of abortion, history of stillbirth, all of them did not present with rash and all of them were multipara.

## DISCUSSION

Viruses such as Human cytomegalovirus (HCMV), Rubella virus (RV) and Herpes simplex type 2 (HSV2) are common cause of unfavourable pregnancy outcomes mostly in low- and middle-income countries (LMICs). This study investigated presence of antibodies to these viruses among women with previous unfavorable pregnancy outcomes in the Lake Victoria zone. In the current study, the HCMV IgG seropositivity was 98% which is significantly higher than 73.9% that was reported among normal pregnant women in the same settings about three years ago.^11^ This could be explained by the fact that, the current study enrolled women with previous unfavorable pregnancy outcomes which signifies the possible role of HCMV in causing adverse pregnancy outcomes in this setting. This has been observed in previous studies whereby abortion, stillbirth and preterm deliveries were significantly associated with HCMV IgG seropositivity.^18, 19^ Further studies to investigate the role of HCMV in relation to previous unfavorable pregnancy outcomes are warranted in this setting.

The observed HCMV seropositivity among women with unfavourable pregnancy outcomes in the current study is comparable to the previous reports in India and Iraq that reported seropositivity of 91.05%, and 96%, respectively.^5,20^ This can be explained by the fact that in the current study the median number of the family size among enrolled women was high which can favor HCMV transmission as previously reported.^21^ In addition, most of women in the current study were found to have low socioeconomic status (SES) which has been associated with high HCMV transmission rates elsewhere.^4, 21^ As previously reported, low SES is often accompanied with poor hygienic and overcrowding conditions which has been found to favor HCMV transmission.^22–24^

In the current study, seropositivity of specific HCMV IgM antibodies was low which is comparable to a previous study in the same setting among normal pregnant women.^11^ However, this observation is different from previous studies in Jordan, and west Iraq which documented IgM seropositivity of 2.3% and 60.2%, respectively.^20,25^ Increased family size has been associated with high HCMV transmission which has been confirmed in the current study whereby median number of household members was high including those who were IgM seropositive. This has confirmed the previous observation whereby children were implicated as sources of HCMV transmission to their parents.^26^ In the current study, due to high seropositivity with almost all population studied being IgG seropositive, associated factors were not studied. This is different from previous studies which observed association between HCMV seropositivity with low SES.^4, 21^ In other similar study in Tikrit, Iraq high seropositivity of HCMV IgM was associated with large family size.^21^

On the other hand, RV seropositivity of IgG was found to be very high which is different from previous studies conducted among pregnant women in Ibadan Nigeria^27^ and Bangladesh^28^ that reported the seropositivity of 68.5% and 84.3%, respectively. This might be due to geographical variation of the seropositivity of RV. In comparison to a previous study in similar setting among normal pregnant women more than 5 years ago, there is no significant differences in IgG seropositivity.^29^ Despite high level of natural immunity among women of reproductive age in the study area^9^, RV might be associated with adverse pregnancy outcomes as reported previously in the same setting.^16^ The seropositivity of RV IgM antibodies among women with previous unfavorable pregnancy outcomes in this study was 1.2% which is comparable to a previous study among normal pregnant women in the same setting.^29^ However, this is inconsistent with previous studies in Karachi, Pakistan that reported seropositivity of 18%.^30^ The factors associated with seropositivity were not investigated due to very high Rubella IgG seropositivity and very low Rubella IgM seropositivity observed in this study. This observation is comparable to the previous studies conducted in Mwanza^29^ and Ibadan Nigeria among a pregnant women.^27^

Regarding HSV-2, IgG seropositivity was found to be slightly low compared to a previous study in Mwanza which reported seropositivity of 34.5% among pregnant adolescent girls.^31^ The possible explanations could be differences in age whereby younger age has been associated with more risk behaviors compared to the population in the current study. In addition, seasonality may also account for differences whereby the current study was conducted from May to August which might be different from previous studies. Seasonal variation has been suggested to be an important factor in HSV-2 transmission elsewhere.^32^ The observed IgM seropositivity in the current study is comparable to a previous report in Nigeria whereby 2.8% of the study participants were HSV-2 IgM seropositive.^33^

Low education level and being married was significantly associated with HSV2 IgG seropositivity. Low education level is often accompanied with poor understanding on the risk factors which can put individuals at risk of acquiring STIs including HSV2. This has been confirmed in the current study whereby more than two thirds of the enrolled women had primary education level. Being married was significantly associated with HSV2 IgG seropositivity in the current study. This is similar to the previous study in Shandong Province China.^34^ As documented in previous report, being married might be accompanied by risk behaviors by partners such as infidelity that might expose individual women to contracting STIs including HSV2.^35^ Factors associated with HSV-2 IgM seropositivity among women with previous unfavorable pregnancy was not computed due to very low HSV-2 IgM seropositivity observed in this study.^36^

In this study we could not establish whether infection of these viruses occurred before, during or after pregnancy therefore this can be considered as one of the limitations. In addition, being the cross-sectional study cannot assess a causality relationship between the exposure and the outcome.

## CONCLUSION AND RECOMMENDATIONS

Compared to RV and HSV-2, IgG seropositivity of HCMV among women with previous unfavorable pregnancy outcomes residing in urban and rural areas of Mwanza region was significantly higher than a previous report among normal pregnant women in the same setting.

### Limitation

The recall bias from the participants and the recruitment included the participants 2 years after having bad pregnancy outcomes and this could have affected our study.
